# Physical Properties and Biofunctionalities of Bioactive Root Canal Sealers In Vitro

**DOI:** 10.3390/nano10091750

**Published:** 2020-09-04

**Authors:** Seung Bin Jo, Hyun Kyung Kim, Hae Nim Lee, Yu-Jin Kim, Kapil Dev Patel, Jonathan Campbell Knowles, Jung-Hwan Lee, Minju Song

**Affiliations:** 1 Institute of Tissue Regeneration Engineering (ITREN), Dankook University, 119 Dandae-ro, Cheonan 31116, Korea; jsbin3000@gmail.com (S.B.J.); jin911031@naver.com (Y.-J.K.); kapildpatel20@gmail.com (K.D.P.); j.knowles@ucl.ac.uk (J.C.K.); 2 UCL Eastman-Korea Dental Medicine Innovation Centre, Dankook University, 119 Dandae-ro, Cheonan 31116, Korea; 3 Department of Conservative Dentistry, College of Dentistry, Dankook University, 119 Dandae-ro, Cheonan 31116, Korea; hyoenk2@naver.com (H.K.K.); lhn2726@gmail.com (H.N.L.); 4 Department of Biomaterials Science, College of Dentistry, Dankook University, 119 Dandae-ro, Cheonan 31116, Korea; 5 Department of Nanobiomedical Science & BK21 PLUS NBM Global Research Center for Regenerative Medicine Research Center, Dankook University, 119 Dandae-ro, Cheonan 31116, Korea; 6 Division of Biomaterials and Tissue Engineering, Eastman Dental Institute, University College London, London WC1E 6HH, UK; 7 The Discoveries Centre for Regenerative and Precision Medicine, Eastman Dental Institute, University College London, London WC1E 6HH, UK

**Keywords:** root canal sealers, bioactive, osteogenic induction, angiogenic, silicate ions

## Abstract

Calcium silicate-based bioactive glass has received significant attention for use in various biomedical applications due to its excellent bioactivity and biocompatibility. However, the bioactivity of calcium silicate nanoparticle-incorporated bioactive dental sealer is not much explored. Herein, three commercially available bioactive root canal sealers (Endoseal MTA (EDS), Well-Root ST (WST), and Nishika Canal Sealer BG (NBG)) were compared with a resin-based control sealer (AH Plus (AHP)) in terms of physical, chemical, and biological properties. EDS and NBG showed 200 to 400 nm and 100 to 200 nm nanoparticle incorporation in the SEM image, respectively, and WST and NBG showed mineral deposition in Hank’s balanced salt solution after 28 days. The flowability and film thickness of all products met the ISO 3107 standard. Water contact angle, linear dimensional changes, and calcium and silicate ion release were significantly different among groups. All bioactive root canal sealers released calcium ions, while NBG released ~10 times more silicon ions than the other bioactive root canal sealers. Under the cytocompatible extraction range, NBG showed prominent cytocompatibility, osteogenecity, and angiogenecity compared to other sealers in vitro. These results indicate that calcium silicate nanoparticle incorporation in dental sealers could be a potential strategy for dental periapical tissue regeneration.

## 1. Introduction

Root canal sealers are widely used dental materials for endodontic treatment. They are applied to fill the space between the core material (i.e., gutta-percha) and the root canal inner wall during the canal filling process to seal off the root canal system, trap the remaining microbes, and pack irregularities in the root canal [1,2]. Key characteristics of materials that are considered for use as root canal sealers include good sealing ability, small film thickness, dimensional stability, insolubility, short setting time, biocompatibility, and so on [3,4].

Among currently available root canal sealers, resin-based sealers represented by AH Plus are the most widely used. Several studies have considered AH Plus as the gold standard, given its excellent physical properties and sealing ability [5,6,7]. Considering that endodontic failure occurs mainly due to bacteria remaining in the canal, functional root canal sealers with antibacterial effects have also been recently investigated as a strategy for better root canal therapy [8,9,10,11,12,13,14,15]. Another improvement in root canal sealers for increasing the success rate of endodontic treatment is the addition of calcium silicate powders to resin-based sealers to accelerate biological functionalities (osteoconductivity or osteoinductivity), which are known as calcium silicate-based sealers [16]. When sealers or their toxic components in extraction are exposed to the surrounding tissues outside the root canal (periapical lesion) [17,18], inflammation of the periapical tissue can be affected and delayed wound healing can result [17,19,20]. Calcium silicate powders are known to have excellent bioactivity, and possibly released components, such as calcium and silicate ions, have been known to accelerate the healing process, along with additional antibacterial effects by alkaline pH [21,22,23,24]. In particular, calcium and silicate ions can accelerate osteoinduction and angiogenesis, respectively, all of which are major biological properties that boost the periapical healing process [25,26,27,28,29]. In addition, when these ions are optimally incorporated in conventional resin sealers, the sealers show comparable physical properties to their counterparts without calcium silicate powders.

Calcium silicate-based sealers, including more reactive calcium-silicate-based bioactive glass, known as bioactive root canal sealers, have been recently introduced [30,31,32]. Bioactive glass has been mainly used for dental hard tissue regeneration; however, it has also recently shown promise for the repair of several complex tissues [33]. The application of bioactive glass to root canal sealers is promising for promoting the regeneration of periapical tissue and achieving predictable clinical outcomes in endodontic treatment due to the greater release of therapeutic ions from bioactive glass.

Nanoparticles have been considered as valuable means in medicinal discipline by the reason of their high surface area to volume ratio and consequence versatility [34,35]. Indeed, nanoparticle-based biomaterials can interact with several biomolecules [36], resulting in being employed to several biomedical areas—e.g., drug delivery, tissue engineering, and dentistry [37]. Although there are several studies applying calcium silicate nanoparticle-based sealers in root canal treatment [38,39], there are few studies exploring the bioactivity of calcium silicate nanoparticle-incorporated sealers.

The aim of this study was to verify the physical properties and biofunctionalities of three commercially available bioactive root canal sealer products (Endoseal MTA (EDS), Well-Root ST (WST), and Nishika Canal Sealer BG (NBG)), which are expected to have calcium silicate nanoparticles, compared with a resin-based control sealer (AH Plus (AHP)). After, acellular bioactivity and various physical properties, such as flowability, film thickness, water contact angle, linear dimensional change, and ion release, were evaluated, and cellular bioactivities in terms of cytocompatibility, cell migration, osteogenic differentiation and angiogenesis were investigated using human periodontal ligament stem cells or human umbilical vein endothelial cells. The null hypothesis of this investigation is that there are no differences in the myriad of physical and biological properties between bioactive root canal sealers and resin-based control sealers.

## 2. Materials and Methods

### 2.1. Materials

Four root canal sealers—namely, AH Plus jet (Dentsply Sirona, York, PA, the United States; AHP), Well-Root ST (Vericom, Anyang, South Korea; WST), Endoseal MTA (Maruchi, Won-ju, South Korea; EDS) and Nishika-BG (Nippon Shika Yakuhin, Shimonoseki, Japan; NBG) were used in this study. The compositions of the sealer materials are described in Table 1.

### 2.2. Surface Morphology and Elemental Composition Analyses

The surface elements of each material were analysed using energy dispersive X-ray spectroscopy (EDS, *n* = 3). All sealers were mixed according to the manufacturer’s instructions, moulded into discs with a diameter of 10 mm and a height of 1 mm, which were set under conditions of 37 °C, 5% CO_2_, and 95% humidity for 72 h to allow complete setting. Then, half of the samples were set in Hank’s balanced salt solution (HBSS) for 28 days, and the other half of the samples were set in deionized (DI) water for 28 days, followed by observation by field emission-scanning electron microscopy (Hitachi, Ltd., Tokyo, Japan: S-4800, FE-SEM) and X-ray energy dispersive analysis (EDX). An accelerating voltage of 20 kV was applied using SEM, and an EDX resolution of 1.0 nm (15 kV)–1.4 nm (1 kV) with 5000× magnification was used. The surface and incorporated nanoparticle morphology of each pre-set (or as-given) sealers are observed under an accelerating voltage of 5 kV using SEM. All samples were coated with platinum for electrical conductivity.

### 2.3. Ion Releasing Profiling

The released ions from the materials were identified using inductively coupled plasma optical emission spectrometry (ICP-OES, Optima 4300 DV, PerkinElmer, Shelton, CT, the United States). The sealer materials were moulded into discs with a diameter of 10 mm and a height of 1 mm, and set under 37 °C and 100% humidity for in 1 h or 72 h. The set materials were then immersed in 20 mL DI water for 4 h and 1, 3, and 7 days, and the solutions were used for leached ion identification (*n* = 3).

### 2.4. Film Thickness Measurement

The film thickness was measured according to the ISO 3107 standard [40]. Two square glass plates with an optically flat surface, an area of 200 mm^2^, and a thickness of 5 mm were prepared and their thicknesses were measured using a digital micrometre (Absolute Digimatic 500-197, Mitutoyo Corp, Kawasaki, Japan). After measuring these two flat glass plates together, the mixed cement of 0.015 g was placed between the glasses. The glasses with cement were pressed with 150 N loading at least for 10 min, followed by measuring the thickness of the glasses with the spread cement. The film thickness of each specimen was considered as the difference between thickness measurement before and after loading. Each group had a sample size of 3 and the mean values of the thickness were calculated.

### 2.5. Flow Distance Measurement

To measure the flow distance of each product, each 0.05 mL sealer was uniformly placed on the plate to create a circle. The second plate was placed, and the sealer was allowed to flow for 10 min at 120 g weight. The flow distance from the smallest and largest diameters was measured using a Vernier calliper (series 530; Mitutoyo, Tokyo, Japan) and averaged as the representative value for each group containing a sample size of 3.

### 2.6. Linear Dimensional Change after Setting

Each sealer was mixed according to the manufacturer’s instructions. Cylindrical moulds with inner diameters of 5 mm and heights of 6 mm were placed on a glass plate covered by a polyethylene plate, and the mixed sealers were placed in the moulds with little overflow. The other glass plate with a polyethylene plate was placed on top of the moulds, and a C-clamp was used to firmly fix the glass plates and moulds. The moulds and clamps with the sealers were transferred to a cabinet at 37 °C and 95% humidity. After the sealer was set, the top and bottom sides of the sealer specimen were polished using 600-grit sandpaper under wet conditions, and the height was measured as the length (*L*0) using a digital calliper with a resolution of 0.001 mm. The specimens (*n* = 3) were stored in distilled water (DW) or HBSS and kept in an incubator throughout the study period (4, 24, 72 h and 7 days). After each assigned period, the lengths of the specimens (*L*) were measured to calculate the percent linear dimensional change (D) using the following equation:

$$D=\frac{L-L0}{L0}\times 100\;\left(\%\right)\;$$

### 2.7. Water Contact Angle (WCA) Analysis

The water contact angle (WCA) of each sample was measured to identify their hydrophilicity. A benchtop phoenix contact angle measurement system (PHX300, SEO, Suwon, South Korea) was employed with the sessile drop method. Each sealer specimen was prepared as a disc shaped with 15 mm diameter and 2 mm height, and approximately 10 μL of DW was dropped onto each sample disc. Each droplet on the sealer specimen was measured 10 s after dropping and automatically recorded by a video recording device. From the collected video data, the surface contact angles were analysed by a Phoenix 300 touch automatic contact angle analyser (Surface Electro Optics Co., Suwon, Korea) (*n* = 3).

### 2.8. Preparation of Sealer Extract Media

All sealers were mixed according to the manufacturer’s instructions and then placed into polyvinyl chloride moulds (inner diameter = 2 mm, thickness = 2 mm), and the excess was carefully removed. The discs containing material were incubated at 37 °C, 5% CO_2_, and 95% humidity for 1 h or 72 h to allow initial or complete setting. After setting, all discs were immersed in α-MEM (Thermo Fisher Scientific, Waltham, MA, the United States) supplemented with 10% fetal bovine serum (FBS; Gibco, Waltham, MA, the United States) and gentamycin (Gibco) at a ratio of 0.5495 cm^2^/10 mL between the surface area of the disc and the volume of medium for 24 h. The extracts were filtered using a 0.22-μm-pore syringe filter (Millipore, Burlington, MA, the United States) and stored at 4 °C until use. The extract media were used at a concentration of 50% in cell experiments.

### 2.9. Isolation and Culture of Human Periodontal Ligament Stem Cells (hPDLSCs)

The experimental protocol was approved by the institutional review board of the Dankook University Dental Hospital, Cheonan, Korea (approval number: DKUDH IRB 2019-01-006). Human PDLSCs were isolated as described in detail in a previous report [41]. Briefly, periodontal ligament (PDL) tissue was carefully dissected from the middle third of the root with a sterilized scalpel and minced into pieces that were as small as possible. The minced PDL tissue was washed 3 times with α-MEM medium containing gentamycin and digested in a solution, including 2 mg/mL collagenase (Gibco) and 4 mg/mL dispase (Gibco) for 1 h at 37 °C. After incubation, the pellets were washed 3 times with α-MEM supplemented with 10% FBS and gentamycin. The cells were seeded in 100 mm dishes and cultured in α-MEM supplemented with 20% FBS, 200 μM L-glutamine (Gibco), 2-mercaptoethanol (Sigma-Aldrich, ST. Louis, MO, the United States) and penicillin/streptomycin (Gibco) at 37 °C, 5% CO_2_, and 95% humidity for 7–10 days to generate cell colonies. The colony-forming cells were considered as hPDLSCs and passed for expansion. Generally, cells at passages 3–5 were used for further experiments.

### 2.10. Cell Viability Assay

A cell counting kit-8 (CCK-8, DOJINDO, Kumamoto, Japan) was employed to evaluate the cytotoxicity of various root canal sealers. Briefly, hPDLSCs were plated in 96-well plates at 2 × 10^3^ cells/well conditions. After 24 h of stabilization, the cells were treated with each sealer extracts for 6, 12, 24, and 72 h. After incubation, 20 μL of CCK-8 solution (water-soluble tetrazolium salt) was added to each well and the plates were incubated for 2 h at 37 °C. The optical density at 450 nm of each well was quantified with iMark microplate reader (Bio-Rad, Hercules, CA, the United States). The percentage of living cells was calculated in comparison to the control group.

### 2.11. Direct Contact Assay

For the direct cell contact cytotoxicity assay, all root canal sealers were mixed and placed on the 60 mm dish surface. The dishes were incubated for 1 h or 72 h at 37 °C, 5% CO_2_, and 95% humidity. After setting, hPDLSCs were directly seeded on sealer specimens at a density of 3 × 10^4^ cells/dish. After 6, 24 and 72 h, the distances between the specimen and the cells were measured in 4 directions from the specimen (*n* = 4).

### 2.12. Inflammatory Gene Expression

Inflammatory gene expression of hPDLSCs was identified under extract media treatment. The hPDLSCs were plated in 6-well plates at 3.2 × 10^4^ cells/well conditions. After 24 h of stabilization, the cells were treated with various root canal sealer extracts for 72 h. After incubation, the total RNA of each sample was isolated with Ripospin^TM^ RNA isolation kit (GeneAll Biotechnology, Seoul, South Korea) and reverse transcription from extracted RNA was conducted using an iScript ^TM^ DNA synthesis kit (Bio-Rad, Hercules, CA, the United States). SensiMix^TM^ SYBR^®^ Hi-ROX Kit (Bioline, London, the United Kingdom) was utilized for Real-time PCR amplification with an AB 7500 Real-Time PCR System (Life Technologies, Carlsbad, CA, the United States). The PCR conditions were as follows: initial incubation at 95 °C for 5 min, followed by 40 cycles of 90 °C for 15 s, 55 °C for 15 s, and 72 °C for 15 s. The primer sequences are listed in Table 2. The relative gene expression levels were calculated using the 2 − ΔΔCt method (*n* = 4).

### 2.13. Osteogenic Differentiation Assay

To identify the osteogenic potential of each material, hPDLSCs were plated in 24-well plates at 5 × 10^3^ cells/well conditions. After reaching 90–100% confluence, the cells were incubated in 1/2 diluted extraction medium containing osteogenic supplements (100 μM ascorbic acid 2-phosphate, 10 mM β-glucerophosphate and 10 nM dexamethasone) for 3, 7, and 21 days. The osteogenic gene expression of hPDLSCs was analysed using the same method described in Section 2.12. The primer sequences are listed in Table 2. For the ALP staining of 3- and 7-day differentiated cells, the cells were rinsed twice with PBS and fixed in 4% paraformaldehyde (PFA) at room temperature (RT) for 20 min. After washing the fixed samples with PBS, the cells were incubated with BCIP/NBT (5-bromo-4-chloro-3-indolylphosphate/nitro-blue tetrazolium) tablets (Sigma-Aldrich, ST. Louis, MO, the United States, #B5655) on an orbital microplate shaker for 5 min. Then, the substrate solution was aspired and rinsed with DW to stop the reaction. For alizarin red staining (ARS) of 21-day differentiated cells, the cells were washed twice with PBS and fixed in 70% EtOH at RT for 10 min. The fixed cells were then washed twice with PBS and 500 μL of 2% ARS solution (pH 4.2) was added to each well and the plates were incubated in the dark at RT for 30 min. Each well was rinsed three times with DW to completely remove the redundant stains. ALP-positive cells and mineralized nodules were imaged with a digital scanner.

### 2.14. Angiogenesis Assay

Human umbilical vein endothelial cells (HUVECs, 2.0 × 10^5^ cells/mL) at passages 4–5 with or without 25% extract were plated together on Matrigel (100 µL) in each well of a 24-well plate for 12 h under complete culture medium composed of vascular cell basal medium (ATCC, PCS-100-030), an endothelial cell growth kit-VEGF (ATCC, PCS-100-041), and 1% penicillin/streptomycin (Gibco, Waltham, MA, the United States). Tubular structure formation (*n* = 5) was visualized using a microscope (Juli Stage, NanoEntek Inc. Guro-gu, Seoul, South Korea), and images were analysed to detect circle and node numbers in a high-power field (hpf) using ImageJ.

Angiogenic gene expression levels of hPDLSCs were investigated through PCR analysis with vascular endothelial growth factor (VEGF), platelet-derived growth factor BB (PDGF-BB), and basic fibroblast growth factor (bFGF) at days 3 and 7, which were analysed via the same method described in Section 2.12. The primer sequences are listed in Table 2. Gene expression data from AHP were excluded due to the low quality of RNA.

### 2.15. Statistical Analysis

Statistical analysis of the data was performed using the Origin Pro 9.0 software package. The results were compared by one-way analysis of variance (one-way ANOVA). When significant differences were found, post-hoc analyses of Scheffe’s adjustment were conducted. *p*-values less than 0.05 were considered statistically significant.

## 3. Results

### 3.1. Physicochemical Properties of Bioactive Root Canal Sealers

**Figure 1:**
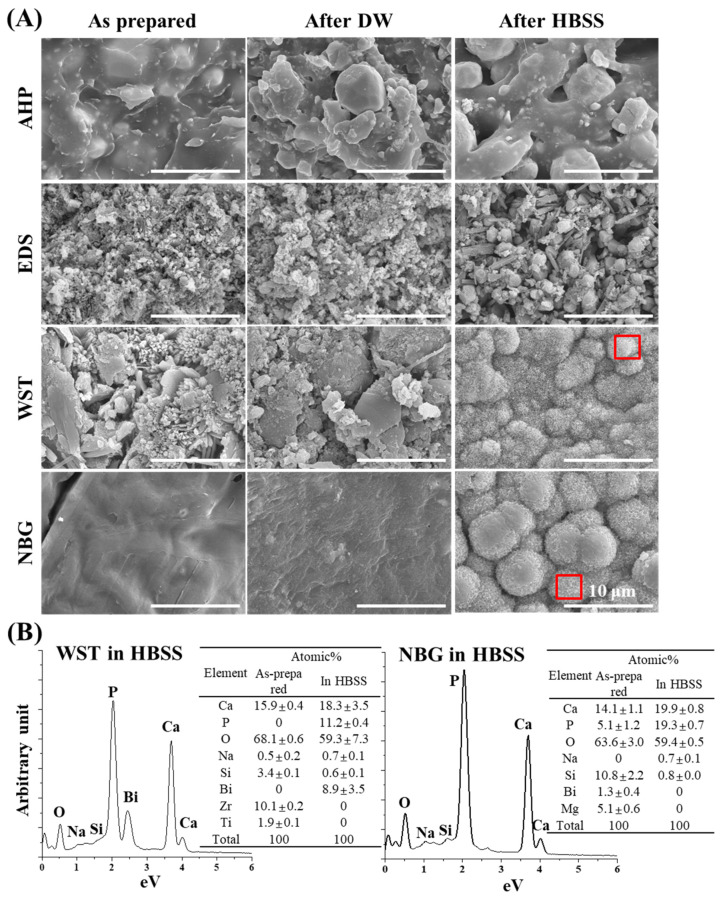
SEM images of the bioactive set sealers before and after DW or HBSS immersion. (**A**) SEM images revealing surface changes of each set sealer between the as-prepared and immersed specimens. Mineralization was observed on the surfaces of HBSS-immersed WST and NBG samples. The red boxes indicate the area analysed with EDS to evaluate the composition. (**B**) The EDS results represent high amounts of Ca and P after HBSS immersion from WST and NBG, indicating calcium phosphate mineralization. Other specific components (Na, Si, and Bi for WST and Na and Si for NBG) were detected in each sealer set.

**Figure 2:**
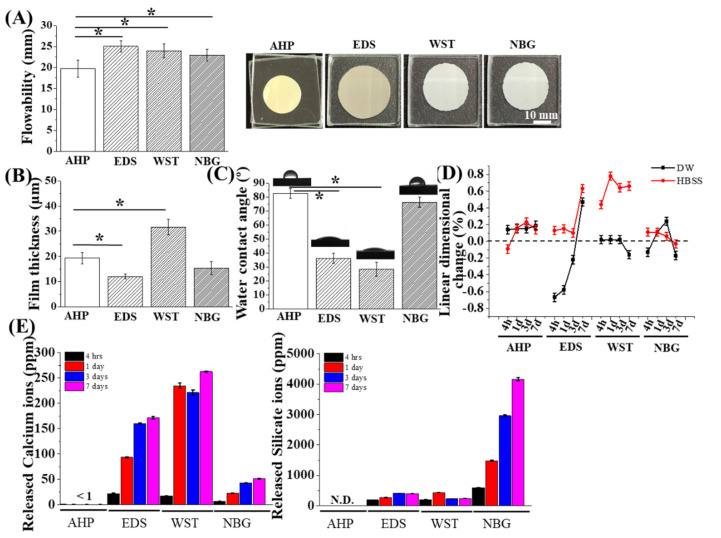
Physical and chemical properties of the bioactive set sealers. (**A**) Flowability of each material. EDS, WST, and NBG showed greater flowability than AHP, while all materials passed the ISO flowability standard (17 mm, *n* = 3). (**B**) The current sealer materials presented film thicknesses under 50 μm, which meet the ISO standard, while EDS showed the thinnest film thickness (*n* = 3). (**C**) The water contact angle of each sealer set presented different values (30–80 degrees) among groups (*n* = 4). (**D**) Linear dimensional change results between DW and HBSS solution over time (*n* = 3). EDS and WST revealed significant differences between DW and HBSS. (**E**) Ion release for 7 days (*n* = 3). EDS and WST exhibited excellent calcium ion release, while silicate ion release was predominant in NBG with time. The asterisks indicate significant differences at the 0.05 level between groups.

The surface morphology of each pre-set (or as-given) sealers are analysed using SEM (Supp. Figure S1). The SEM images of as-given sealers revealed that EDS and NBG contain 200 to 400 nm and 100 to 200 nm particles, respectively, while AHP and WST do not, implicating the existence of nanoparticles in as-given EDS and NBG. Next, each sealer material was prepared as solid discs after setting and their surface morphology and elemental compositions were verified by SEM and EDS, respectively (Figure 1). The resin-based sealer AHP showed little difference between the as-prepared sample and immersed samples (Figure 1A). In contrast, the bioactive root canal sealers—i.e., EDS, WST, and NBG—showed distinct changes in surface morphology, especially in WST and NBG. The globular mass of each specimen predominantly contained Ca and P, but also contained other elements—i.e., O, Na, Si, and Bi. The flowability, film thickness, WCA, and linear dimensional change in each material were also measured (Figure 2A–D). All the materials showed the flowability over 17 mm, the film thickness under 50 µm, and the WCA from 30 to 80 degrees. Regarding the linear dimensional change, WST showed changes between the specimen immersed in DW and HBSS, while the other materials showed little change.

The calcium and silicon ion release profile of each material was also identified (Figure 2E). The exudate of the resin-based AHP sealer contained few calcium and silicate ions, while the exudates of the other bioactive root canal sealers showed an increase in ion release tendency over time. In all bioactive root canal sealers, silicate ions exhibited greater release than Ca. In EDS, 2.3 times more silicate ions were released than Ca, and in WST, 1.1 times more silicate ions were released. However, for NBG, 0.2 to 0.3 times less Ca was released from NBG than from the other two bioactive root canal sealers, whereas 4156 ppm silicate ions were released from NBG, corresponding to 80 times more silicate than Ca from NBG, 10 times more silicate than Ca from EDS and 18 times more silicate than Ca from WST.

### 3.2. Cytocompatibility of hPDLSCs to Bioactive Root Canal Sealers

**Figure 3:**
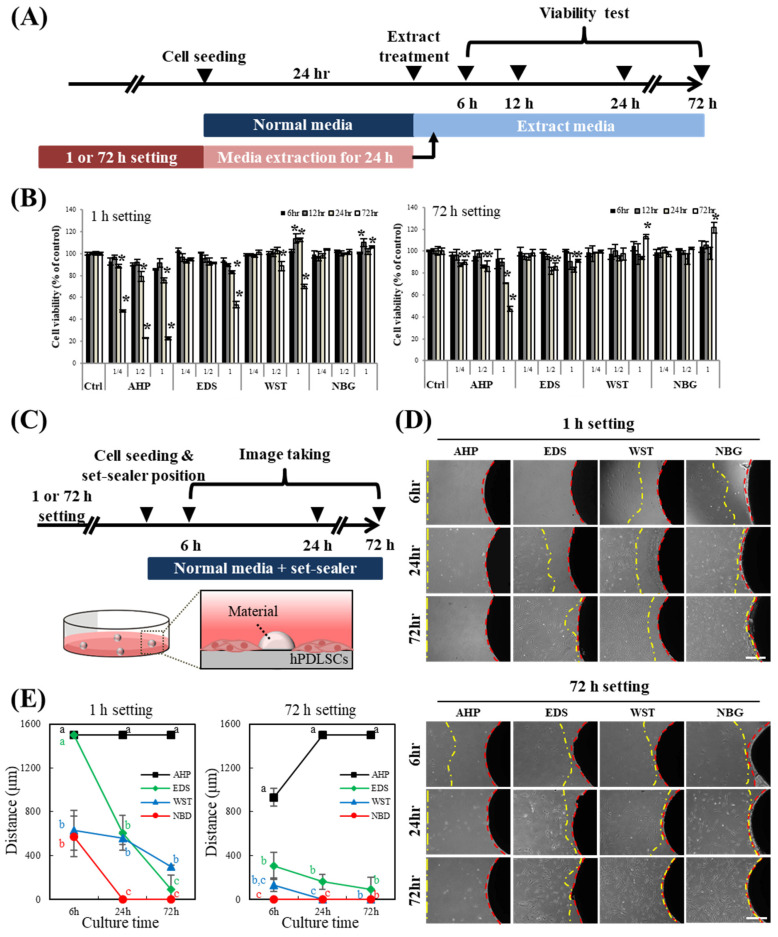
Biological characteristics of the bioactive set sealers. (**A**) Schematic timeline of the cell viability assay with the sealer extract in media. (**B**) Cell viability assay of hPDLSCs with set sealer extract or its dilute for up to 72 h. The extract media of 1- or 72-h-set-AHP showed severe cytotoxicity to hPDLSCs both at the original extract concentration and at 50% dilution, while the other extract media showed acceptable viability (~90%) under 50% dilution. Asterisks indicate significant differences compared to each control (*n* = 5, *p* < 0.05). Increased cell viability was observed for WST and NBG. (**C**) Schematic timeline of the direct contact assay with the set sealers. hPDLSCs were cultured directly with the set sealers. (**D**,**E**) Cells migrated towards the 1- or 72-h sealers over time. hPDLSCs cultured with bioactive root canal sealers (EDS, WST and NBG) migrated and made contact with the materials, while cells rarely migrated toward the set-AHP. Cells cultured with NBG displayed the fastest migration among the groups. Different letters indicate significant differences among the groups in each time point (*n* = 4, *p* < 0.05).

The cell viability and direct interaction with each root canal sealer material were determined (Figure 3). Even with 1 h sealer setting and extraction, bioactive root canal sealers showed nearly 100% cell viability to hPDLSCs until 72 h if cultured at 50% dilution, while one-hour-set AHP exudate showed cell viability below 50% at 72 h of culture, even in 25% dilution of the exudate (Figure 3A,B). AHP, with 72 h of setting, showed cell viability over 80% at 72 h of culture. A direct contact assay was also conducted with hPDLSCs and root canal sealers (Figure 3C–E). At 6 h of culture with the one-hour-set materials, hPDLSCs more closely approached WST and NBG than AHP and EDS (Figure 3D,E), and at 72 h of culture, the cells had migrated very close to all of the materials except AHP. When cultured with 72-h-set materials, all bioactive root canal sealers showed reasonable cytocompatibility with hPDLSCs over time, while the cells did not approach the resin-based AHP sealers.

**Figure 4:**
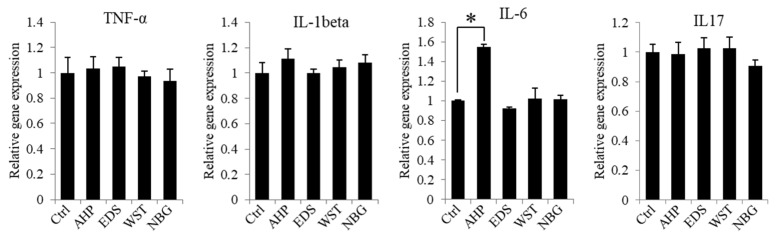
Inflammatory and anti-inflammatory gene expression of hPDLSCs in set sealer extracts. hPDLSCs showed no change in inflammatory (TNF-α, IL-1β and IL-6) or anti-inflammatory (IL-17) gene expression in 50% diluted extract from EDS, WST and NBG after 1 hr of setting, while AHP from 72 h of setting showed significant upregulation of inflammatory gene expression (IL-6). The asterisk indicates a significant difference between groups (*n* = 3, *p* < 0.05).

Inflammatory and anti-inflammatory gene expression of hPDLSCs was identified while culturing with the root canal sealer extract in vitro (Figure 4). AHP extract upregulated the expression of interleukin 6 (IL-6), which is a pro-inflammatory gene, while the AHP extract did not upregulate the other pro-inflammatory genes—i.e., IL-6, tumour necrosis factor alpha (TNF-α) or interleukin 1 beta (IL-1β), and interleukin 17 (IL-17). The bioactive root canal sealers did not increase the expression of inflammatory genes compared with the control.

### 3.3. Osteogenic Differentiation Capacity of Bioactive Root Canal Sealers

**Figure 5:**
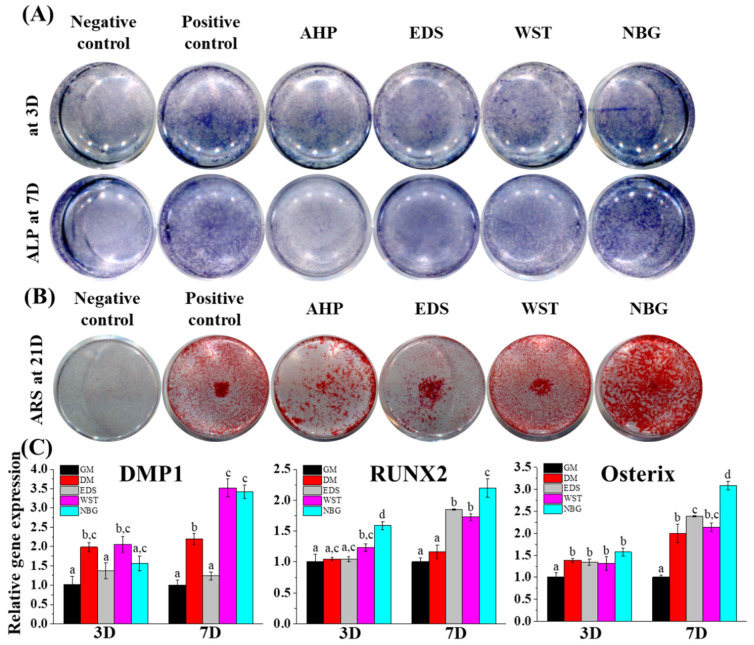
Osteogenic differentiation effects of the bioactive set sealers. (**A**,**B**) hPDLSCs cultured with basal media (negative control), osteogenic media (positive control), or sealer extract media (50%) revealed that both alkaline phosphatase (ALP, violet colour) activity and Alizarin red S (ARS, red colour) staining were highest in the NBG group at early (days 3 and 7) and late (day 21) differentiation stages. (**C**) Osteogenic gene expression (DMP1, RUNX2, and osterix) from extract (50% dilution) was upregulated in the bioactive root canal sealers, while NBG showed the highest expression among groups at days 3 and 7. Gene expression data from AHP were excluded due to low quality of RNA. Different letters indicate significant differences among the groups (*n* = 3, *p* < 0.05).

The osteogenic potential of each root sealer was analysed in vitro (Figure 5). After hPDLSCs were cultured for 3 and 7 days with osteogenic media only (positive control) or sealer extracts in osteogenic media, early osteogenic capacities were determined by alkaline phosphatase (ALP) staining (Figure 5A) and related gene expression (Figure 5C). NBG showed the highest ALP expression compared with the other groups. Consistently, NBG highly upregulated runt-related transcription factor 2 (RUNX2) on both days 3 and 7. On day 7, the WST- and NBG-treated groups showed significant upregulation of dentin matrix protein 1 (DMP1) and RUNX2 compared with the groups treated with osteogenic media. The EDS-treated group showed a significant increase in RUNX2 and osterix (OSX) on day 7. The hPDLSCs were cultured under the aforementioned conditions for 21 days to identify the final osteogenic differentiation with ARS staining, which showed calcium deposition (Figure 5B). The WST group showed as much calcium deposition as the positive control group, while the NBG group showed higher calcium deposition than even the positive control. The EDS and AHP groups showed less osteogenic tendency than the positive control group.

### 3.4. Angiogenic Capacity of Bioactive Root Canal Sealers

**Figure 6:**
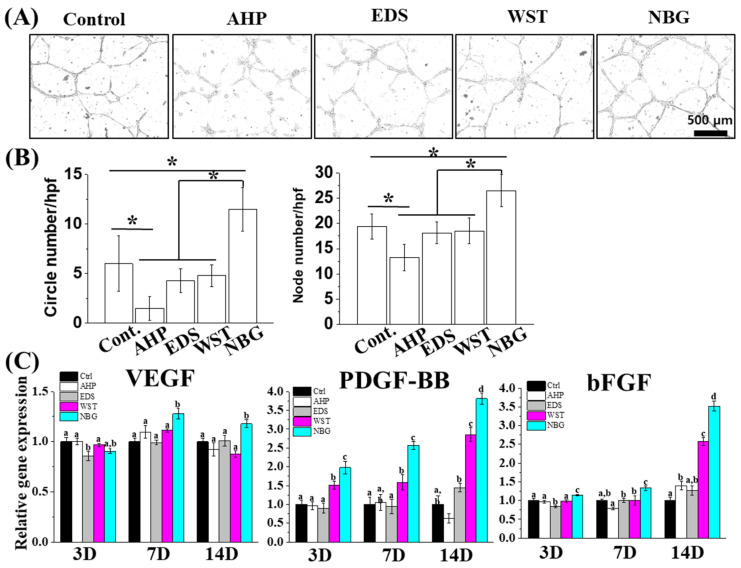
Angiogenic effects of the bioactive set sealers. (**A**,**B**) HUVECs cultured with basal media or sealer extract (25% dilution) revealed that both circle and node numbers were highest in the NBG group compared with the other groups. Asterisks indicate significant differences among the groups (*n* = 3, *p* < 0.05). (**C**) Angiogenic gene expression of hPDLSCs over 14 days from extract after 50% dilution revealing that angiogenic gene (VEGF, PDGF-BB and bFGF) expression was the most upregulated in the NBG group. Different letters indicate significant differences among the groups (*n* = 3, *p* < 0.05).

The angiogenic potential of each sealer extract medium was verified by a tube formation assay with HUVECs and by angiogenic gene expression profiling with hPDLSCs. The bioactive root canal sealers showed distinguished tube numbers and node numbers compared with AHP. Among these bioactive root canal sealers, NBG-extract media-treated HUVECs presented the highest degree of tube and node formation (Figure 6A,B). Consistently, the WST and NBG sealer groups showed trends toward increased PDGF-BB and bFGF at days 7 and 14 in hPDLSCs. Notably, the NBG group exhibited 1.2–4 times higher expression of angiogenic genes at every time point except VEGF on day 3 (Figure 6C).

## 4. Discussion

The null hypothesis of this investigation—that there are no differences in the myriad of physical and biological properties between bioactive root canal sealers and resin-based control sealers—was rejected based on current results, which revealed differences in cell toxicity, migration, osteogenic differentiation, and angiogenesis. We studied whether nanobioglass-incorporated bioactive root canal sealers have proper physicochemical and biological properties. Because root canal sealers are materials that are used in the final stages of root canal therapy, HBSS immersion conditions were set to roughly mimic the sealer-exposed situation to the biological environment of the apical foramen. When the root canal sealer was immersed in DW after setting, there was no marked change on the surface of the material compared with the as-prepared material. When immersed in HBSS, a composition of which is more similar to that of body fluid than DW, the surfaces of the bioactive root canal sealers showed a roughened aspect with an amorphous calcium phosphate-like structure, which was commonly displayed by the other bioactive surface (Figure 1A) [26,27,42,43,44]. Considering the Ca/P atomic ratios of WST and NBG after HBSS immersion were different to each other (WST: 1.63 and NBG: 1.03, according to the Figure 1B), there are also some possibilities that the surfaces of these samples contain several types of calcium phosphate of which Ca/P ratio varying from 0.5 (Monocalcium phosphate monohydrate and monocalcium phosphate anhydrous) to 2.2 (Amorphous calcium phosphate: 1.2–2.2) [45], which will be further evaluated with high-resolution X-ray diffraction analysis or Fourier-transform infrared spectroscopy. More precise analysis about changes in functional groups or chemical bonds, as well as quantitative ratios among surface elements, could be evaluated by or X-ray photoelectron spectroscopy in future study [26,27,46,47]. These data are consistent with another finding, which revealed that ions—i.e., calcium and silicate ions—are released from these materials (Figure 2D).

According to ISO 3107, the flowability should be over 17 mm and the film thickness should be under 50 μm, and these standards are met by all the current root canal sealers (Figure 2A,B). The WCA data showed that all the bioactive root canal sealers exhibited similar or prominent hydrophilicity compared with AHP, which may be relevant to the biocompatibility [48]. Linear dimensional changes in EDS and WST showed an expanded tendency when immersed in HBSS. It is possible that this change could hamper the sealing capacity or that internal stress from this change could reinforce the sealing effect. Considering these results with the fact that AHP and calcium-silicate based sealers can adhere to the dentin wall and improve the sealing effect [49,50,51,52], these sealers are viewed as compliant materials in mechanical aspects.

The aesthetic properties of the material comprise one of the issues considered in dental clinics. We did not evaluate the discolorations of each sealer by the reason that most of the dental sealers, including AH plus and calcium silicate based sealers, are reported to raise tooth discoloration [53,54]. Although the mechanical and biological properties should be considered prior to aesthetic properties, the improvement in discoloration issues should be considered in further study for the patient’s quality of life.

The ion release tests revealed that all the bioactive root canal sealers showed more calcium release than AHP, while NBG predominantly released 10–18 times more silicate ions than the other bioactive root canal sealers, suggesting the possible enhancement of biological properties due to the release of therapeutic ions. Together, these bioactive root canal sealers showed reasonable physicochemical characteristics for root canal sealers, while releasing calcium and silicate ions.

Although AHP is a widely used root canal sealer because of its low cytotoxic effects compared with original resin-containing sealers (i.e., AH-26) [55], epoxy resin-based sealers are known for their tentative toxicity by remaining monomers [56,57]. In this regard, bioactive root canal sealers can be a good alternative considering the issue of biocompatibility while releasing biorelevant ions [58]. The cytocompatibility of the bioactive root canal sealers was identified with a cell viability assay and a direct contact assay under two different time conditions (1 h and 72 h) (Figure 3). Many studies have performed biological tests with 48-h setting conditions of the sealer, which is based on the international standard ISO10993-12. However, in the clinical environment, a freshly mixed endodontic sealer is applied to the canal and will be exposed to surrounding periapical tissue. Therefore, a 1-h setting condition of current sealers was used in this experiment to ensure a more clinically relevant study. As expected, AHP showed a certain degree of cell toxicity, while the other sealers showed eminent cytocompatibility. In particular, the direct contact assay, which mimics the clinical situation in which the periodontal ligament cells contact the sealers on the apex, revealed that bioactive root canal sealers led to greater migration of hPDLSCs to the surrounding set specimen and consequent better biocompatibility from the early stage than widely used resin-based sealers. Among the bioactive root canal sealers, calcium silicate nanoparticle-containing NBG showed the most prominent cytocompatibility and migration ability for hPDLSCs, even compared to another calcium silicate nanoparticle-containing EDS. In addition to the fact that EDS showed not much change in its surface when immersed in both DW or HBSS, these results might be due to the different amount of bismuth in each bioactive root canal sealer, as was observed on the surface of HBSS-immersed WST. Indeed, Collado-González et al. reported low cell proliferation, low viability, and restricted attachment of hPDLSCs with EDS [59]. Another report also revealed that EDS showed high levels of aluminium and contained bismuth [60], which are typical compositions of Portland cement and are also found in HBSS-immersed WST, but not NBG. Even though the material safety data sheet (MSDS) of NBG reports that it contains bismuth (Table 1), the EDS data of HBSS-immersed NBG showed no bismuth, which might be the reason for our direct and indirect toxicity data (Figure 3).

Root canal therapy is performed to prevent or heal apical periodontitis and to fill apical lesions with healthy bone and accompanying vessels [61]. Calcium ions and silicate ions are known to enhance osteogenic differentiation, and silicate ions can stimulate angiogenesis [25,26,27,28,29], which can be released from current bioactive root canal sealers (Figure 2D). The media extract with bioactive root canal sealers showed predominant early and late osteogenic potentials to hPDLSCs compared with the resin-based extract (Figure 5). Human PDLSCs, in addition to human dental pulp stem cells, are key stem cell types that regenerate hard tissue at the surrounding apex (end of the root) after root canal treatment [62,63]. Among the materials tested, NBG showed the most intense ALP staining, the highest osteogenic gene expression, and the most extensive ARS staining. Considering the aforementioned results that NBG released the most silicate ions (Figure 2D), silicate ions, rather than calcium ions, seem to play a more critical role in osteogenesis. We further examined the angiogenic capacity of each material with a tube formation assay using endothelial cells and evaluated the accompanying angiogenic gene expression profiles of hPDLSCs (Figure 6). Consistent with the ion release results, NBG not only formed the largest number of tubes and nodules but also showed the highest expression of angiogenic genes, which might also result from silicate ion release.

The above data suggest that NBG not only enhanced osteogenic and angiogenic gene expression but also stimulated both osteogenesis and angiogenesis while maintaining the necessary physical characteristics of a sealer material. It is expected that the synergistic effect of several physicochemical and biological properties of NBG could reduce microleakage of the apical lesion when NBG is applied in clinical situations. Thus, NBG and other bioactive root canal sealers could possibly be widely used sealers in dental clinics.

## 5. Conclusions

Here, we analysed several bioactive root canal sealers to determine their mechanical, chemical, and biological properties in comparison to the resin-based root canal sealer. The bioactive root canal sealers, especially nanoparticle-incorporated NBG and nanoparticle-absent WST, showed active surface transitions followed by biorelevant ion release, especially calcium and silicate ions. The extract media from bioactive root canal sealers showed low toxicity, intense osteogenic capacity, and high angiogenic potential with respect to hPDLSCs and HUVECs, while the sealers maintained the appropriate mechanical properties for use as root canal sealers. Among the tested bioactive root canal sealers, nanoparticle-incorporated NBG presented the most prominent biological capacity, mainly due to its abundant silicate ion release from its nanoparticle, rather than calcium ion release, compared with the other sealer materials. These results suggest that NBG and other bioactive root canal sealers have the potential to be used as root canal sealer materials in dental clinics.

## Figures and Tables

**Table 1:** The root canal sealer materials tested in this study.

Product	Code	Manufacturer	Lot Number	Composition (wt%) *
AH Plus	AHP	Dentsply	1910000640	Component A: epoxyresin (25–50%), calcium tungstate, zirconium oxide, aerosol, iron oxide
				Component B: adamantane amine, N,N-dibenzil -5-oxanonane, TCD-diamine, calcium tungstate, zirconium oxide, aerosol
Endoseal MTA	EDS	Maruchi	CD191207C	Calcium silicates (dicalcium silicate), tricalcium aluminates, calcium aluminoferrite, calcium sulfates, bismuth oixide, zirconium oxide (66.5%), thickening agent
Well-Root ST	WST	Vericom	WR9N6100	Calcium silicate compound, calcium sulfate dehydrate, calcium sodium phosphosilicate, zirconium oxide, titanium oxide, thickening agents
Nishika-BG	NBG	Nippon shika yakuhin	H7T	Component A: fatty acid, bismuth subcarbonate, silicon dioxide
				Component B: magnesium oxide, purified water, calcium silicate glass, silicon dioxide

* The compositions were according to the available information included in the manufacturer’s material safety data sheets.

**Table 2:** The primers used for polymerase chain reaction analysis.

Gene Name	Sequence (5′ → 3′)
GAPDH_Fwd	CCAGAACATCATCCCTGCCTCT
GAPDH_Rev	GACGCCTGCTTCACCACCTT
TNF-alpha_Fwd	CGTGGAGCTGGCCGAGGAG
TNF-alpha_Rev	AGGAAGGAGAAGAGGCTGAGGAAC
IL-6_Fwd	GGTGTTGCCTGCTGCCTTCC
IL-6_Rev	GTTCTGAAGAGGTGAGTGGCTGTC
IL-1beta_Fwd	TGGCTTATTACAGTGGCAATGAGGATG
IL-1beta_Rev	TGTAGTGGTGGTCGGAGATTCGTAG
DMP-1_Fwd	CAGGAAGAGGTGGTGAGTGAGT
DMP-1_Rev	TGGATTCGCTGTCTGCTTGCT
RUNX2_Fwd	TCCAGACCAGCAGCACTCCATA
RUNX2_Rev	TCCATCAGCGTCAACACCATCA
OSX_Fwd	CAGCAGCTAAACTTGGAAGGA
OSX_Rev	TGCTTTCGCTTGTCTGAGTC
IL17_Fwd	TCAACCCGATTGTCCACCAT
IL17_Rev	GAGTTTAGTCCGAAATGAGGCTG
VEGF_Fwd	CAAAAACGAAAGCGCAAGAAA
VEGF_Rev	GCGGGCACCAACGTACAC
PDGFBB_Fwd	CTGGCATGCAAGTGTGAGAC
PDGFBB_Rev	AATGGTCACCCGAGTTTGG
bFGF_Fwd	GGCTTCTTCCTGCGCATCCA
bFGF_Rev	GCTCTTAGCAGACATTGGAAGA

## Supplementary Materials

The following are available online at https://www.mdpi.com/2079-4991/10/9/1750/s1, Figure S1: Scanning electron microscopy images of each sealer.
