# Supplementary material for: Physical Properties and Biofunctionalities of Bioactive Root Canal Sealers In Vitro
Source: Nanomaterials (Basel). 2020 Sep 4;10(9):1750. doi: 10.3390/nano10091750 (PMC7559325; doi:10.3390/nano10091750)
Supplement: Supplementary file 1 [file nanomaterials-10-01750-s001.pdf]

Physical Properties and Biofunctionalities of Bioactive Root Canal Sealers In Vitro

Seung Bin Jo [1,2,†], Hyun Kyung Kim [3,†], Hae Nim Lee [3], Yu-Jin Kim [1,4,5], Kapil Dev Patel [1,2,5], Jonathan Campbell Knowles [1,2,6,7], Jung-Hwan Lee [1,2,4,5,*] and Minju Song[1,3,*]

[1] Institute of Tissue Regeneration Engineering (ITREN), Dankook University, 119 Dandae-ro, Cheonan 31116, Korea; jsbin3000@gmail.com (S.B.J.); jin911031@naver.com (Y.-J.K.); kapildpatel20@gmail.com (K.D.P.); j.knowles@ucl.ac.uk (J.C.K.)

[2] UCL Eastman-Korea Dental Medicine Innovation Centre, Dankook University, 119 Dandae-ro, Cheonan 31116, Korea

[3] Department of Conservative Dentistry, College of Dentistry, Dankook University, 119 Dandae-ro, Cheonan 31116, Korea; hyoenk2@naver.com (H.K.K.); lhn2726@gmail.com (H.N.L.)

[4] Department of Biomaterials Science, College of Dentistry, Dankook University, 119 Dandae-ro, Cheonan 31116, Korea

[5] Department of Nanobiomedical Science & BK21 PLUS NBM Global Research Center for Regenerative Medicine Research Center, Dankook University, 119 Dandae-ro, Cheonan 31116, Korea

[6] Division of Biomaterials and Tissue Engineering, Eastman Dental Institute, University College London, London WC1E 6HH , UK

[7] The Discoveries Centre for Regenerative and Precision Medicine, Eastman Dental Institute, University College London WC1E 6HH, London, UK

* Correspondence: ducious@gmail.com (J.-H.L.); minju81s@dankook.ac.kr (M.S.); Tel.: +82-41-550-3081 (J.-H.L.); Tel.: +82-41-550-1943 (M.S.); Fax: +82-41-559-7839 (J.-H.L. & M.S.)

† Contributed equally to this work as first authors.

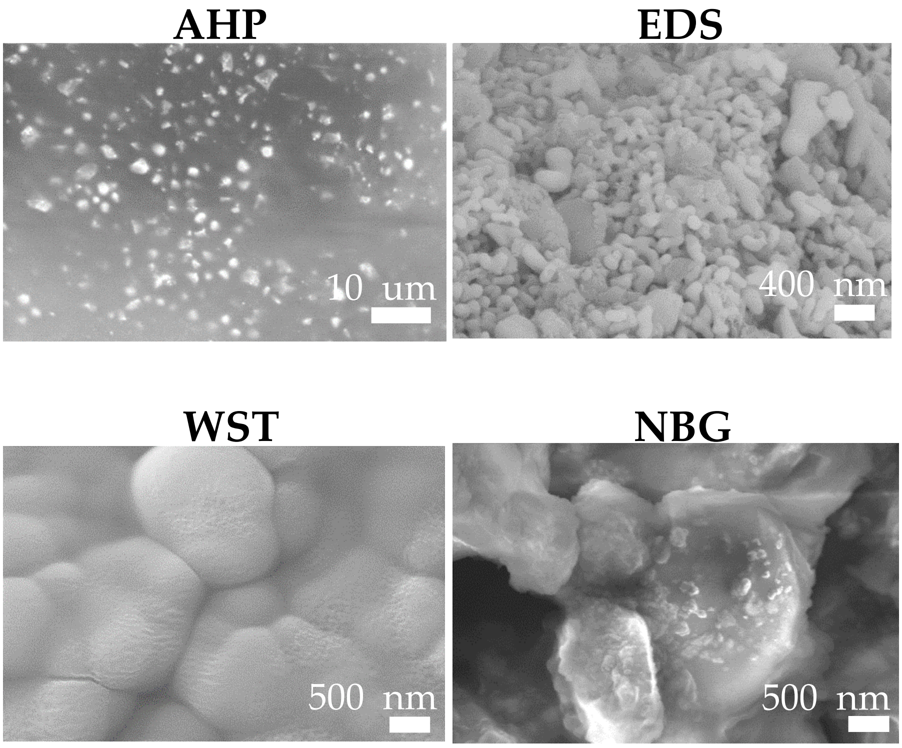

Supplementalry Figure 1. Scanning electron microscopy images of each sealer. EDS and NBG showed incorporated particles of 200–400 nm and 100–200 nm size, respectively. Images of AHP and WST revealed their smooth surface, indicating absence of nanoparticle in these sealers.
